# An innovative multimorbidity patient-centered care model in Chile: implementation evaluation results

**DOI:** 10.1186/s13690-025-01516-4

**Published:** 2025-05-09

**Authors:** Jaime C. Sapag, Mayra Alicia Martínez Pérez, Paula Zamorano, Teresita Varela, Paulina Muñoz, Romina Seguel, Esteban Irazoqui, Álvaro Téllez

**Affiliations:** 1 School of Public Health & School of Medicine, Department of Family Medicine), Faculty of Medicine, Pontificia Universidad Católica de Chile, 11 Santiago, Chile; 2 Dalla Lana School of Public Health, University of Toronto, Toronto, Canada; 3 Institute for Mental Health Policy Research, Centre for Addiction and Mental Health, Toronto, Canada; 4 Centro de Innovación Áncora UC, Pontificia Universidad Católica de Chile, Santiago, Chile; 5 Centro de Innovación en Salud Áncora UC, Facultad de Medicina, Pontificia universidad Católica de Chile, Santiago, Chile; 6 Health Technology Assesment Unit, Center of Clinical Research, Pontificia Universidad Católica de Chile, Santiago, Chile; 7 Department of Family Medicine, Pontificia Universidad Católica de Chile, Santiago, Chile

**Keywords:** Multimorbidity, Chronic care, Implementation evaluation, Primary health care, Health care innovation, Latin America, Chile

## Abstract

**Background:**

The impact of non-communicable diseases and multimorbidity challenges health systems worldwide. Latin America faces an urgent need to develop practical innovations in that regard. The Centro de Innovación en Salud ANCORA UC implemented a new Multimorbidity Patient-Centered Care Model (MPCM) pilot in Chile between 2017 and 2020. MPCM aimed to reorganize health services from a fragmented diagnosis-based perspective towards a new approach based on patient’s needs and offer intervention strategies according to their multimorbidity risk. This article aims to report the evaluation of the implementation process of MPCM in the Southeast Metropolitan Health District in Chile.

**Methods:**

The study design corresponds to an implementation collaborative evaluation of MPCM innovation using qualitative methodology. Two main questions guided the research: (1) How has MPCM been implemented in its pilot phase? Moreover, (2) What are the main learnings from the MPCM pilot phase and their contribution to its scalability at the national level? In addition, the *Consolidated Framework for Implementation Research* and the *Outcomes for Implementation Research* were considered in the theoretical approach.

**Results:**

Thirty-five (35) interviews were conducted with 69 professionals and key stakeholders involved in the implementation process of MPCM, including health practitioners, transition nurses who coordinate the intervention with the affiliated hospitals, managers, and the implementation team. Overall, the results were positive, suggesting that a complex innovation of this kind may be implemented successfully. Key lessons learned should be considered for scaling up MPCM to the national level. Some critical barriers to implementation were high staff turnover and the COVID-19 pandemic, while leadership and team commitment were relevant facilitators.

**Conclusions:**

This study represents a new step in evaluating an innovative model for addressing multimorbidity in Chile. The scaling up phase requires careful consideration of all lessons learned, as well as a robust evaluation and monitoring plan. This research represents the first evaluative analysis of MPCM in the context of a complex innovation adapted to enhance public health policies using implementation evaluation approaches. *Implementation Science* is a fundamental approach to fostering quality improvement strategies for health care in Latin America.

**Table:** 

Text box 1. Contributions to the literature
This study presents the implementation evaluation of an innovative Multimorbidity Patient-Centered Care Model (MPCM) developed and scaled up in Chile.
The *Consolidated Framework for Implementation Research* (CFIR) and *Implementation Outcomes* were considered to analyze the multiple barriers and facilitators affecting the development of MPCM.
Qualitative findings suggest a successful implementation of a pilot process at a Health District level, identifying lessons learned and concrete recommendations relevant for scaling up.
This extensive, comprehensive evaluation represents a concrete example of using CFIR to better understand and enhance a complex intervention to address multimorbidity in Latin America and worldwide.

## Background

Chronic non-communicable diseases and multimorbidity are the most significant challenges for health systems worldwide [1, 2]. In the Americas, they are responsible for almost four out of every five deaths annually, and this is likely to increase in the coming decades due to demographic and epidemiological changes [3–5]. Multimorbidity is a real issue in Latin America, where about 20–40% of adults over 18 years old declare having at least two conditions [6]. Preventing and addressing multimorbidity is now a key priority, and work is being developed to build effective and sustainable models of care [7].

Multimorbidity is associated with greater disability and mortality, worse quality of life, and more frequent use of health care services [8, 9]. It increases with age and is more common among women and individuals of lower socioeconomic status [10]. One chronic disease is often related to developing other chronic diseases, implying further complexity to chronic disease management [3, 11]. Today’s health systems need to be better prepared to address this challenge. Hence, they need a new configuration of their services, levels, and sectors to deliver care that can efficiently and equitably address multimorbidity [1].

Complex health interventions present a major organizational, structural, and operational challenge [12]. The feasibility, as well as the implementation, are critical aspects that deserve to be addressed by clinical teams and decision-makers. Thus, timely adjustments could facilitate the expected success and its long-term sustainability. Various methods have been described to carry out the implementation [13]. However, evaluating the implementation process and its degree of progress requires a deeper effort that presents substantial challenges in the real context [14, 15]. Therefore, evaluation’s critical information undoubtedly facilitates the permanence of changes and their potential scalability to other jurisdictions.

International evidence shows that health systems can effectively face multimorbidity when they have a person-centered approach and work as part of an integrated network [16]. In Latin America, various practices have been developed to address chronicity with promising results, in which self-management, stratification, and shared decision-making are considered essential elements [6]. However, appropriate management of multimorbidity remains a substantial gap [17–19].

Implementing the Family and Community Health Model in Chile has been promoted as a holistic and modern strategy [20, 21]. However, it has yet to break down the fragmented approach to patients with chronic diseases by coexisting multiple vertical programs [22]. The Ministry of Health has published support guidelines for the Cardiovascular Health Program, emphasizing the implementation of a multimorbidity approach and other self-management strategies [23]. Nevertheless, more is needed to manage multimorbidity adequately [24].

In this context, the Multimorbidity Person-Centered Care Model (MPCM) emerges because of the collaborative work between the Centro de Innovación en Salud ANCORA UC (CISAUC), the National Health Insurance Fund (FONASA) and the Servicio de Salud Metropolitano Sur Oriente (SSMSO) [25, 26]. MPCM aims to respond to the challenge of multimorbidity by proposing an appropriate approach for the local context, considering best practices and international evidence.

**Fig. 1:**
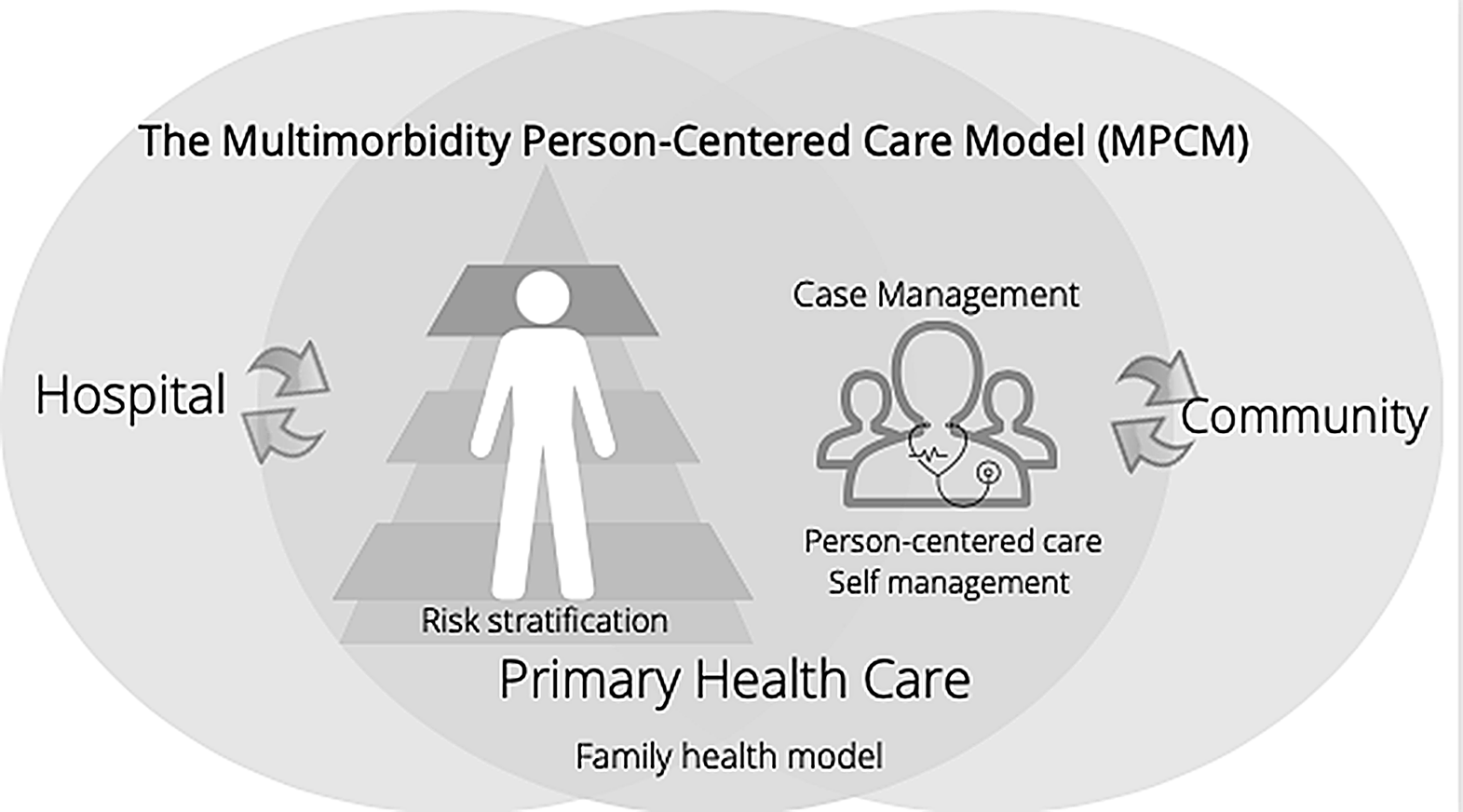
The multimorbidity person-centered care model (MPCM)

MPCM (Fig. 1) is a strategy that organizes the population with multimorbidity according to their complexity through a pyramid approach where specific interventions are assigned according to their needs. The interventions are designed so that those who already have a chronic disease can self-manage them and do not become complicated and that those who already have multimorbidity and some complications can receive adequate and coordinated services. The model is based on Primary Health Care and incorporates essential elements such as self-management, Participation, shared responsibility, case management, continuity of care in the network, and risk stratification [25, 26].

The MPCM was run in the SMSSO from the joint initiative with CISAUC. Among the 29 health districts, SSMSO is among the biggest health districts in Chile. It provides public services in the context of three subnetworks and seven municipalities in the southeast of the Chilean Metropolitan Region under the umbrella of the Ministry of Health [27]. About 1.5 million inhabitants (almost a quarter of the Metropolitan Region population) are in the assigned territory, and around 75% have public insurance. More than 40 primary care centers (Centros de Salud Familiar -CESFAMs-), four hospitals, and various specialized outpatient public facilities exist. A recent pilot study of the impact of MPCM showed that intervened patients had a significantly lower incidence of mortality (OR 0.56; 95% CI 0.40–0.77) compared with regular care [28].

An evaluation and scalability plan for the model is developed for its implementation throughout the health service territory to replicate it successfully and subsequently transfer the experience to the national level. Today, the MPCM has been a fundamental contribution to the Strategy that the Ministry of Health has begun to implement in the rest of the country as a “Comprehensive Care Strategy focused on Patient for the Promotion, Prevention, and Management of Chronicity in the context of Multimorbidity” [25].

The MPCM innovation development and its progressive scalability require careful consideration of the implementation processes, given that it is a complex change that affects different areas of the organization and the operation of the care network. It is there where a promising intervention can fail and not achieve the expected impact. Therefore, *Implementation Research* [29], which considers the complexity of these processes, is of particular help in planning and evaluating MPCM and its components. Implementation Science contributes to understanding an intervention in terms of what, why, and how it works or not in this case, the MPCM works (or does not) in real contexts. In addition, an implementation evaluation may help to identify possible ways to improve.

This article’s objective is to report on the evaluation of the implementation process of MPCM in the Southeast Metropolitan Health District.

## Methods

### Design

This study design is a collaborative evaluation of an innovative intervention using qualitative methodology. Two main questions guided the research: (1) How has MPCM been implemented in its pilot phase? and (2) What are the main learnings from the MPCM pilot phase and their contribution to its scalability at the national level? The Standards for Reporting Implementation Studies (StaRI) were used to frame this study and report its findings [30].

### Participants

The selection of participants was carried out through convenience sampling and snowballing. The inclusion criteria considered individuals who had performed functions associated with the care of the population with multimorbidity in different areas of MPCM: (1) decision-makers and management teams, (2) health teams, and (3) officials of organizations linked to MPCM that were part of the process of implementation and consolidation of the health network. Thirty-five (35) interviews were conducted with health professionals and key stakeholders involved in the implementation process of MPCM at SSMSO, including professionals from CESFAM pilots and referral hospitals, transition nurses who coordinate the intervention with the corresponding hospitals, managers, and the implementation team of the CISAUC and SSMSO.

### Data collection

The data collection process started once authorization was obtained from the territorial health authorities and each related organization. It was carried out between October and December 2020. Interviews were scheduled in coordination with local MPCM implementation coordinators at the health centers and directly with the participants in the case of individual interviews. Interviews were conducted remotely and lasted about 60 min each. They were recorded and later *verbatim* transcribed for analysis. A total of 13 individual and 22 group interviews were conducted. Of those included, 29 were made to multidisciplinary health teams in primary care, two to transition nurses from the three referral hospitals, two to decision-makers from the SSMSO, and two to the CISAUC team. As a result, the data collection process concluded with high levels of data saturation.

In-person data collection was canceled because of the COVID-19 pandemic. To protect the health and safety of respondents and research staff, most of the interviews were conducted remotely through Zoom’s communications platform.

### Theoretical framework for the evaluation

**Fig. 2:**
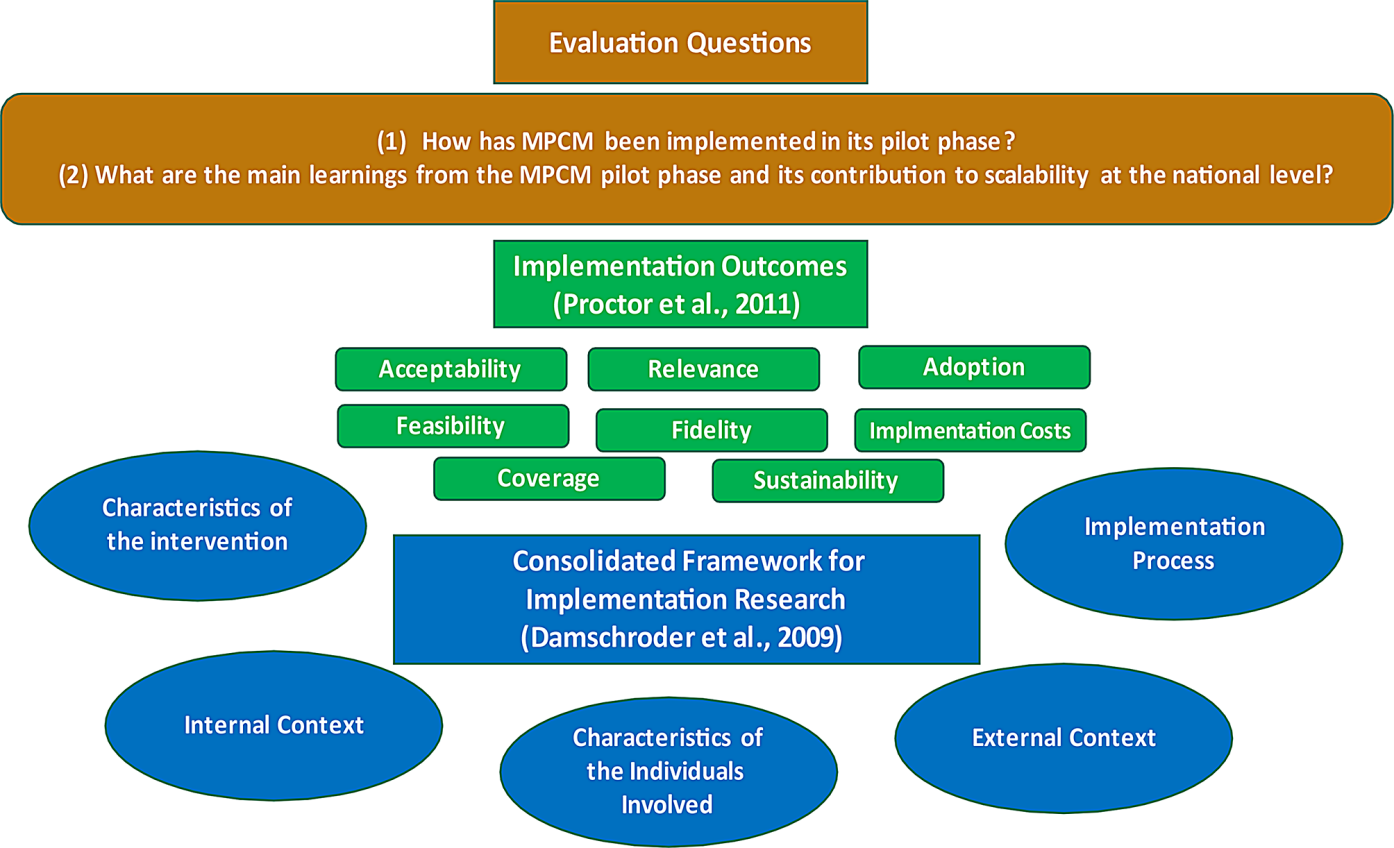
Integrated Framework for Implementation Evaluation of the Multimorbidity Person-Centered Care Model (MPCM)

Two main theoretical frameworks were synergically used (Fig. 2): the *Consolidated Framework for Implementation Research* (CFIR) [31, 32] and the so-called *Outcomes for Implementation Research* [33]. CFIR identifies five central areas relevant to implementation, including 39 constructs and sub-constructs: (1) Characteristics of the intervention, (2) Internal Context, (3) External Context, (4) Characteristics of the Individuals Involved, and (5) Implementation Process. The framework can be used to guide implementation assessments, evaluate implementation progress, and explain findings in research studies [31, 32]. This study used CFIR in data analysis to identify emerging factors that influenced implementation.

Complementarily, *Outcomes for Implementation Research* [33] were considered to serve as success indicators of what happened to the implementation results in terms of (1) acceptability, (2) adoption, (3) relevance, (4) feasibility, (5) fidelity, (6) implementation cost, (7) coverage (penetration) and (8) sustainability.

### Analysis

Content analysis [34] was conducted, attending to the evaluative questions and considering the CFIR and *Implementation Outcomes* as a guide. This made possible to classify and group the information into specific categories to account for key properties and dimensions.

Open coding facilitated the possible construction of relationships between categories with sufficient information, seeking content saturation to obtain information that validated these relationships and complemented the categories that enriched the refinement and development.

The information analysis process was carried out considering the rigor criteria of qualitative research [35]. The first interviews were conducted, and these were verbatim transcribed. The general analysis was carried out in each one to incorporate new information that was pertinent to contrast with other participants in the following interviews.

Additionally, an interpretative triangulation of the data was carried out to increase the reliability and quality of the selection of the coded interview segments. On the one hand, investigator triangulation was conducted, where two researchers independently reviewed each of the transcripts and selected segments according to the main categories. Subsequently, these analyses were contrasted to ensure relevance. Additionally, theoretical triangulation was carried out using the Consolidated Framework for Implementation Research [31, 32] and the Outcomes for Implementation Research [33] to obtain a more holistic understanding from the Implementation Sciences perspective.

### Ethics

#### Ethical approval

Ethical approvals were obtained at both Pontificia Universidad Católica de Chile and SSMSO (#200717004: “Evaluación de la Implementación y Satisfacción Usuaria en el Modelo de Atención Centrado en la Persona con Morbilidad Crónica (MACEP), en el Servicio de Salud Metropolitano Sur Oriente”).

## Results

Sixty-nine participants were interviewed (Table 1): 52 women and 17 men. Regarding their roles, 58 were at the seven Pilot CESFAMs (16 in decision-making or managerial roles), three staff at the reference hospitals, and 8 were part of the overall implementation team (CISAUC and SSMSO). All participants are health professionals or technicians and/or have training in the direction and management of health programs.

**Table 1:** Participants roles, location and interview type

Role	Number of persons (%)	Location	Type of Interview	
			Individual	Group
CESFAMs Decision-makers and management team	16 (23%)	PHC	10	3
Health care teams	42 (61)	PHC	2	14
Transition coach nurses	3 (4)	Hospitals	1	1
Overall Implementation team	8 (12)	CISAUC and SSMSO	1	3
Total	69 (100)		14	21

** PHC: Primary Health Centers; CISAUC: Centro de Innovación en Salud ANCORA UC; SSMSO: Servicio de Salud Metropolitano Sur Oriente

Findings are organized following CFIR main dimensions and Outcomes for Implementation Research, focusing more on some relevant subdimensions based on research questions. In addition, a summary of the main perceived implementation gaps is presented.

### CFIR dimensions

**Fig. 3:**
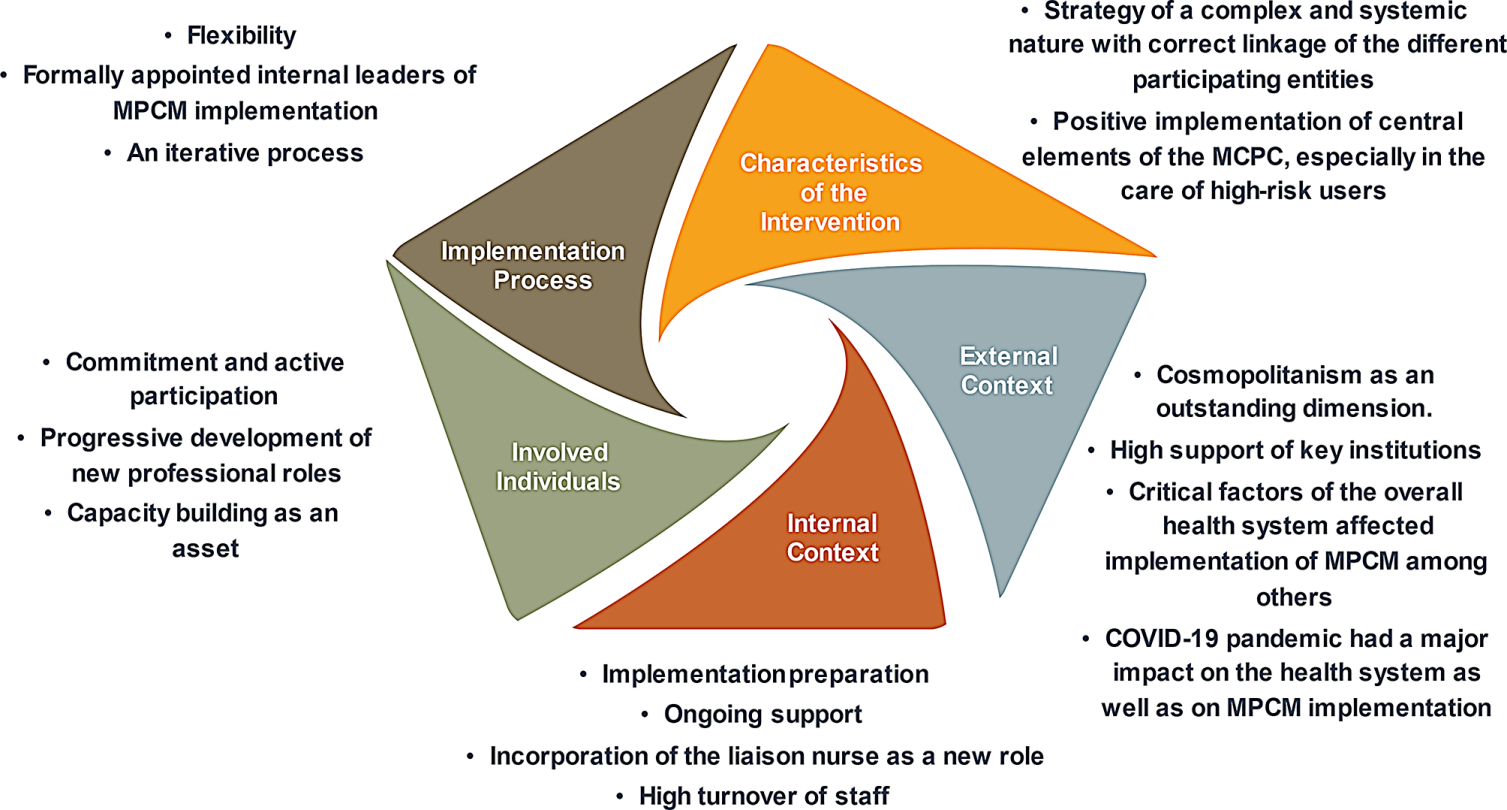
Summary of Main Evaluation Findings According to the CFIR dimensions

Figure 3 summarizes the main findings according to the five dimensions of the CFIR framework.

### Intervention characteristics

Participants considered MPCM a relevant strategy with a complex implementation, which implied linking and articulating different entities to achieve the expected changes in care and the correct adoption of its central elements (Stratification by risk, Continuity of care throughout the network, Self-management support, Case management, Participation, and Shared responsibility).

They assumed the *intervention source* was internal, as it came from the SSMSO itself, but each site had an ongoing adoption process. A particular value of MPCM for patients was identified, which might be more evident for high-risk patients. In a fragmented health system context, the intervention approach was mentioned as *a relative advantage*, with two outstanding elements: Case management and Stratification by risk.

*…they (patients) felt the difference of moving from the model of cardiovascular control to this model…*,* especially the case management; patients felt a great change in care…Because we investigated other pathologies: we did not focus only on hypertension or diabetes… They were really surprised with the type of care… which was a totally different approach. They felt more satisfied*,* more listened to; they felt the change*,* actually. (ES*,* M4*,*91)*

### External context

*Cosmopolitanism* was identified as an outstanding dimension. Fostering existing alliances of managers and decision-makers from the institutions involved, such as Fondo Nacional de Salud (FONASA, which is the National Health Fund), SSMSO, and Community and local Directorates, facilitated a collaborative design of MPCM and its subsequent implementation. Participants agreed that changes in clinical care management were necessary to optimize the relationship between patient needs and resource allocation. Other perceived critical factors of the overall health system that affected the implementation of MPCM were limited resources, rigid roles of health workers, and long waiting times, among others. In addition, during the last year of the MPCM pilot, the COVID-19 pandemic had a significant impact on the health system as well as on MPCM implementation.

*So*,* currently*,* there is a very critical period of our management and of course -within the priorities - we are ´full Covid´*,* so the times have been lengthening… there we also have a super weak point in management. (EI*,* A1*,*73)*

### Internal context

Interviewees indicated that committed executives adequately articulated economic and human resources, facilitating the planning and execution of MPCM. This was considered fundamental for supporting readiness for implementation and the ongoing consolidation of the intervention at different levels of the health network. Being able to enhance networks and communication with other levels and incorporating the transition nurse as a new role were two aspects highly valued by all the participants. One of the identified challenges regarding internal context was the high turnover of health professionals at the participant health centers who were facing their daily challenges.

*…the organization was also going to change. Because of MPCM*,* but also other needs…we changed. There was rotation; somebody left*,* two new nurses arrived*,* and another professional moved. We had to resume the process of new induction. The result of the high turnover we had last year; there were always many changes. (EL*,* M4*,* 5)*

### Characteristics of individuals

High commitment and active participation of the individuals involved, including leaders and executors, in the different units of the health network were recognized as another element that facilitated strategy implementation at the local level. Participants identified the progressive development of specific roles to lead the implementation process, as well as the motivation and involvement of the teams, as critical assets. The capacity-building component builds up the initial human capital needed to execute the activities and actions associated with the change in the care process. This element was highly valued and helped to foster self-efficacy.

*I was in the trainings… I found that they were super good*,* but afterward*,* the way of implementing them in our team was not the best*,* so I think that if all that had been established from the beginning (training)*,* it would have been very different. (ES*,* M4*,*10)*

### Process

According to participants, the implementation process was successfully carried out, keeping the main elements of MPCM but making adjustments according to the local reality of each of the CESFAMs. That was perceived as another element that conditioned the implementation, mainly in its initial phase. Furthermore, the existence of formally appointed internal leaders of MPCM implementation who acted as champions was valued. In addition, the ongoing support—expressed by frequent visits, telephone calls, and meetings—was perceived as relevant for engagement.

*Beginning to install the change in the organization*,* in the teams*,* in the form of care*,* is also accepting certain insecurities by part of the team with some issues that they did not handle… I believe there was also key support from the team…*,* for change management*,* being able to listen*,* contain*,* help set limits*,* or ask for help. Without this accompaniment*,* taking on so much flight (with MPCM) is difficult because there were many concerns… Let us go slowly; you do not have to do everything in a first consultation. It is still a challenge today to prioritize health problems because users come with multiple needs*,* and how to rank them and how to see what goes first*,* what goes next is a constant. (D*,* A3*,*17)*

### Outcomes of implementation

Table 2 summarizes the main findings regarding *the Outcomes of Implementation*. Participants had a positive impression of acceptability, adoption, relevance, and fidelity. Feasibility was more challenging, as it also depends greatly on local realities. However, ongoing resources and support facilitated implementation, so costs must always be considered when scaling up. Regarding fidelity, perceptions were more positive when the focus was on high-risk patients, where the intervention was more intense and started earlier. Low and middle-risk patients constitute a more extensive population, so it helps to reach higher coverage, but it might still imply some fidelity challenges.

**Table 2:** Evaluation findings: outcomes of implementation

1. Acceptability: participants were motivated to incorporate the proposed changes regarding the MPCM in their practices and considered them appropriate within the care system, validating the implementation of the strategy.	
2. ***Adoption***: collected evidence indicates high levels MPCM integration in the health teams, involving the expected different activities for the care of users, following the guidelines provided according to the risk stratification (high, moderate and low complexity).	
3. ***Relevance***: the perception regarding this outcome of implementation was positive, indicating that MPCM is important to improve care for users with multimorbidity. In addition, positive health and quality of life results for the target population were identified.	
4. ***Feasibility***: although participants revealed the possibility of carrying out the changes proposed by MPCM, some differences were identified according to each local reality. Elements that conditioned the viability of MPCM implementation are primarily of an organizational nature, among which the following stand out: (A) modifications in performance to carry out comprehensive patient control, (B) changes in the scheduling system to carry out continuity of care by the head team, (C) an efficient registration system that facilitates the entry of integrated and non-fractioned information by pathology, and (D) protected time to perform associated tasks for the implementing leaders; they considered it advisable to use between 9 and 11 h a week for this function. These organizational elements were modified in a different way by each CESFAM.	
5. ***Fidelity***: the information collected shows a correct implementation of the core elements of MPCM, according to the original guidelines. This is more accurate for high-risk users where MPCM has been fully implemented. Health professionals involved in actions and activities aimed at medium and low complexity considered that they are still in the process of incorporation. They indicate that the high demand for patients and the modification of certain organizational conditions (induction plan, higher yields, changes in schedules, training of personnel, as well as spaces for dissemination and follow-up with those involved) are fundamental for the consolidation of MPCM.	
6. ***Cost***: there is a call to maintain the resources, both financial and human, considered in the pilot stage of the Strategy, to be able to continue progressing in the implementation of MPCM. The most relevant identified resources were the transition nurse and support staff for the clinical management of patients, as well as specific materials such as cell phones, among others.	
7. ***Coverage***: a good level of population scope of the intervention was achieved. Initially with high risk and then with moderate and low risk patients. Progressively, this coverage was greater. However, as the population outreach broadens, significant challenges arise to ensure fidelity in the implementation of the intervention.	
8. ***Sustainability***: participants report a successful institutionalization of MPCM, as it has been established within the pilot CESFAMs. Main mentioned changes are aimed at: (A) comprehensive patient care, (B) delivery of support for individual and group self-management (pre-pandemic), as well as (C) case management, and (D) participation and shared responsibility visualized in the agreed plans and its continuous updating. These changes are mainly consolidated in the attention to highly complex users. Regarding moderate and low complexity, this sustainability process has made great progress that has generated results, but the need to continue with training and dissemination actions.	

Regarding sustainability, which is critical for potential scaling up, interviewees had a positive perception. The following citations are illustrative:

*In the end*,* the greatest changes are noticed in the treatment with patients*,* in the treatment that one has*,* in user satisfaction*,* that is what has occurred a little more*,* that patient when solving various difficulties in the same control. They are happier*,* and as they leave happier*,* adherence improves*,* so it is a chain of benefit*,* and - little by little - the team coordinators have understood it*,* (GC y T*,* M3*,* 36)*

*It was quite a change for the team in different aspects. A lot has to do with performance because now we no longer speak*,* for example*,* of ‘cardiovascular control’*,* but rather ‘chronic control*,*’ and we - at least - talk about that; the patient comes for chronic control*,* and there is no ‘cardiovascular disease’ as previously mentioned. (ES*,* A2*,* 38)*

### Implementation gaps

The information obtained shows some aspects that require further development to improve the implementation of MPCM from the participants’ perspective. They identified the needs to:

- Generate a participatory process to adjust the Strategy, addressing the differentiating elements and challenges according to the Stratification by Risk approach.
- Develop a continuous evaluation plan for the implementation process and monitoring actions that allow timely identification of gaps for sustained improvement. Health teams, especially those in charge of implementation, highlighted this aspect, which indicates that it would facilitate the planning of goals and identification of achievements.
- Having in place a systematic process of training, dissemination, and participation instances that foster the adoption and loyalty of the Strategy was a central aspect noted as necessary for its sustainability throughout the network.
- The individuals involved in the change process go through periods of adaptation that generate certain professional burnout, so having self-care team activities represents an instance that can make a favorable difference in the implementation process from the perspective of the health teams.
- The MPCM Strategy has yet to promote the integration of the mental health component in the comprehensive control of users. This requires specialized training initiatives from an interdisciplinary approach for their integration into care.
- Promote the dissemination of MPCM to the external user (patients) so that the change in the process is bidirectional, which would enhance the results of the Strategy.

## Discussion

Integrating evidence-based innovations - such as MPCM - is difficult, as complex changes challenge health systems, clinical teams, and decision-makers [36]. Therefore, evaluating their implementation is crucial [13, 14, 37]. The current article presented the findings of its implementation evaluation. Overall, the results were positive, indicating that a complex innovation of this kind may be implemented successfully, and critical lessons learned should be considered for scaling up MPCM to the national level.

Other experiences have been developing new models of care for chronic diseases. Previous studies confirmed the importance of identifying the facilitators and barriers to their implementation [38]. Our evaluation found some similarities as well as differences between them.

It is critical to ensure appropriate resources are in place to support change [39]. That includes funding for employing additional chronic care staff, incentives regarding recruiting and retaining healthcare providers [36], and the need to pay special attention to working conditions [40]. In our study, staff turnover – related to internal and external contexts - clearly represents a barrier to implementing MPCM appropriately. This has been a challenge in many other innovation developments [41]. Understanding how turnover affects implementation would contribute to defining a better planning process.

Regarding Continuity of care, communication and coordination challenges between primary care and hospitals are still issues. MPCM makes that evident and starts a change process to ensure integrated work. However, fragmented health systems take time to move further in that direction, but it is possible to enhance impact [42].

Leadership was identified as an ingredient for the success of implementing MPCM in its pilot phase. In particular, the role of transition nurses represents a critical innovation that facilitates MPCM success, concrete leadership [43], and articulation among health services for high-risk users. Strategic leadership development is essential when scaling up MPCM to the national level. Frameworks such as LEAD [44] can contribute to incorporating an explicit approach in that regard.

In addition, team commitment and collaboration at all levels were considered fundamental to reaching MPCM positive results. Other authors have identified effective multidisciplinary teams as an engine for quality improvement in health care [41]. A recent study [45] concluded that promoting team commitment and preventing intra-group conflicts contribute to resilient teams that cope with challenges more easily. Organizational culture is a fundamental element for the potential integration of innovations [46]. Scaling up MPCM at the national level requires preparing healthcare organizations to integrate it into practice.

It is also relevant to consider how the COVID-19 pandemic has affected the implementation of MPCP. Different participants identified it as a barrier to developing the intervention. However, even when it affected the process of implementing MPCM, it did not stop it. As other studies have shown, healthcare utilization significantly decreased because of the pandemic, with considerable consequences for patients [47], requiring high levels of resilience from health services to address that impact [48]. MPCH also had to react and adapt to those actual circumstances.

This study has some limitations. It was considered just a qualitative perspective from staff implementing MPC, but it did not incorporate patients´ voices directly. In order to mitigate that, interview questions invited staff to consider users´ reality. In addition, following rigor standards for qualitative research [49, 50] - such as credibility, transferability, dependability, and confirmability - contributed to minimizing potential biases. For instance, using a solid theoretical framework, considering triangulation in data analysis, and reaching data saturation were relevant. In addition, following STARI guidelines [30] for conducting and reporting this study was an asset.

Implementation research helps address the challenges of the know-how gap in real-world settings and the logistics needed to achieve national and global health goals [51]. There is a need to advance faster in connecting *Implementation Research* with health services development in Latin America [52]. Critical barriers to embedding research into planning and practice in Latin America include policy implementation timeframes and complex political processes. In contrast, some facilitators are the actionability of findings and relevance of implementation research questions [53]. All these elements play a critical role in MPCP implementation and must be appropriately addressed.

## Conclusions

The present study highlights that the evaluation of implementing an innovation like MPCM can identify key aspects from Implementation Science that contribute to pinpointing areas for improvement for national-level scalability. Collaboration between managing and funding institutions is significantly valued as a central contextual aspect. The intervention features targeting patients with high complexity are perceived as particularly beneficial for managing patients with multimorbidity; however, the intervention design for patients with moderate and low complexity should be revisited in order to enhance outcomes. Fragmentation of care was positively addressed through implementing additional roles to support care coordination. The external team’s work from CISAUC with each team of the primary care centers was crucial in adapting the design to local realities, significantly influencing implementation outcomes. This study represents the first evaluative analysis from implementation science of MACEP in the context of complex innovations tailored to enhance public health policies.

## Data Availability

Data availability is not possible for ethical reasons. If you need more information, please contact Jaime C. Sapag (jsapag@uc.cl), the implementation evaluation leader.

## Abbreviations

CESFAMs: Centros de Salud Familiar
CFIR: Consolidated Framework for Implementation Research
CISAUC: Centro de Innovación en Salud ANCORA UC
FONASA: Fondo Nacional de Salud
MACEP: Modelo de Atención Centrado en la Persona con multimorbilidad
MPCM: Multimorbidity Person-Centered Care Model
NCD: Non-Communicable Diseases
PHC: Primary Health Care
SSMSO: Servicio de Salud Metropolitano Sur Oriente
